# Understanding the underlying molecular pathways by which *Mboat7/Lpiat1* depletion induces hepatic steatosis

**DOI:** 10.1016/j.jlr.2021.100047

**Published:** 2021-02-11

**Authors:** Federica Tavaglione, Nozomu Kono, Stefano Romeo

**Affiliations:** 1 Department of Molecular and Clinical Medicine, Sahlgrenska Academy, University of Gothenburg, Gothenburg, Sweden; 2 Clinical Medicine and Hepatology Unit, Department of Internal Medicine and Geriatrics, Campus Bio-Medico University, Rome, Italy; 3 Department of Health Chemistry, Graduate School of Pharmaceutical Sciences, The University of Tokyo, Tokyo, Japan; 4 Clinical Nutrition Unit, Department of Medical and Surgical Science, Magna Graecia University, Catanzaro, Italy; 5 Department of Cardiology, Sahlgrenska University Hospital, Gothenburg, Sweden

**Keywords:** AA, arachidonic acid, LKO, hepatocyte-specific KO, *MBOAT7*, *membrane-bound O-acyltransferase domain–containing 7*, NAFLD, nonalcoholic fatty liver disease, PI, phosphatidylinositol, Scap, SREBP cleavage-activating protein, SREBP-1c, sterol regulatory element–binding protein-1c

Nonalcoholic fatty liver disease (NAFLD) is becoming the leading cause of chronic liver disease worldwide, paralleling the global epidemic of obesity and type 2 diabetes (1). In addition to the well-established metabolic and environmental risk factors, a body of evidence supports genetic predisposition as a pivotal driver of NAFLD development and progression to its life-threatening complications, namely cirrhosis and hepatocellular carcinoma. To date, several genetic *loci* have been identified contributing to NAFLD. Noteworthy, the majority of these genetic variations are located in genes involved in lipid biology, including *PNPLA3*, *TM6SF2*, *membrane-bound O-acyltransferase domain–containing 7* (*MBOAT7*), and *HSD17B13* (2).

The common sequence variant rs641738 C>T near the *MBOAT7* gene confers increased susceptibility to NAFLD and the entire spectrum of its conditions by downregulating *MBOAT7* expression in the liver (3). In a recent meta-analysis of 42 studies including more than one million participants, this common variant has been firmly associated with the presence and severity of NAFLD in European adults (4). Interestingly, homozygotes for very rare and severe loss-of-function mutations in *MBOAT7* display severe cognitive impairment with neurodevelopmental defects (5), showing how common and rare genetic variants in the same *locus* may lead to extremely diverse phenotypes.

*MBOAT7* encodes lysophosphatidylinositol acyltransferase 1, a 472-amino acids–long protein with six transmembrane domains present on the ER, lipid droplets, and mitochondria-associated membranes (3, 6). The pronounced effect on the nervous system may be due to an alteration of protein trafficking, a key intracellular pathway for nervous system development. MBOAT7 is involved in the acyl-chain remodeling of membrane phospholipids in the Lands' cycle. Specifically, MBOAT7 has a lysophospholipid acyltransferase activity thought to preferentially incorporate arachidonic acid (AA; 20:4 n-6) into phosphatidylinositol (PI) (3, 6, 7).

In the past year, several lines of research have focused on unraveling the molecular mechanism(s) by which *MBOAT7* deficiency induces hepatic steatosis. In this issue of the Journal of Lipid Research, Xia *et al.* (8) elegantly showed that hepatocyte-specific *Mboat7* depletion caused an increased liver fat content (mostly triglycerides and cholesterol esters) and liver damage (i.e., higher transaminases) in mice fed a chow diet with a fasting-refeeding regime. In addition, by using MS, authors analyzed in detail the phospholipid composition in the liver, showing that the concentration of PI 38:4(18:0_20:4), the most abundant PI in the liver, was decreased by approximately 50% in *Mboat7* hepatocyte-specific KO (LKO) mice. Similarly, the concentration of other PI species containing 20-carbon polyunsaturated fatty acids, including PI 36:3 (16:0_20:3), PI 38:2 (18:0_20:2), and PI 38:3 (18:0_20:3), was markedly decreased in the *Mboat7* LKO liver. Conversely, *Mboat7* depletion led to increased hepatic levels of PIs containing monounsaturated or polyunsaturated without 20-carbon fatty acids, including PI 34:1 (16:0_18:1), PI 36:2 (18:1_18:1), and PI 40:6 (18:0_22:6).

In this work, *Mboat7* depletion induced hepatic steatosis by increasing de novo lipogenesis driven by the activation of sterol regulatory element–binding protein-1c (SREBP-1c), a key lipogenic transcription factor involved in fatty acid biosynthesis. In agreement, the mRNA expression and synthesis of SREBP-1c target genes was found to be increased in the *Mboat7* LKO liver. In addition, the hepatocyte-specific depletion of both *Mboat7* and *SREBP cleavage–activating protein* (*Scap*) normalized hepatic fat content similarly to *Scap* depletion alone, supporting that *Mboat7*-mediated hepatic steatosis was due to SREBP-1c processing. The strength of this study resides in the detailed and accurate lipidomics with lipid flux analysis in genetically engineered *Mboat7* LKO mice.

Serendipitously, a recent study in a similar mouse model showed remarkably consistent results (9). In agreement with the study by Xia *et al.*, Tanaka *et al.* showed that hepatocyte-specific *Mboat7* depletion spontaneously caused liver fat accumulation in LKO mice (9) with increase in the triglyceride content. However, this study (9) did not see any differences in the cholesterol amount, although the trend was in the same direction as the study by Xia *et al.*, indicating a potential lack of power for detecting changes in cholesterol in the study from Tanaka *et al.*

In agreement with the study by Xia *et al.*, the study by Tanaka *et al.* (9) reported similar changes in PI composition in the *Mboat7* LKO liver. Indeed, the authors showed that the hepatic amount of AA-containing PI (PI 38:4) was dramatically reduced in *Mboat7* LKO mice. However, the total amount of PIs was found to be slightly decreased in the LKO liver, whereas it was significantly increased in the study by Xia *et al.* The changes in the levels of other PI species containing monounsaturated and polyunsaturated fatty acids were similar in both studies.

In addition to the mouse model, Tanaka *et al.* (9) investigated the impact of *MBOAT7* depletion on hepatic fat content and PI composition in cultured human hepatocytes, obtaining results similar to in vivo experiments. Within this context, the authors robustly demonstrated that *MBOAT7* deficiency in vitro resulted in hepatic fat accumulation specifically by upregulating triglyceride synthesis, without affecting either triglyceride degradation or secretion. The authors proposed a non-canonical pathway underpinning the association between *MBOAT7* deficiency and hepatic steatosis. Indeed, they suggested that the depletion of AA-containing PI in hepatocytes caused simultaneously an increased PI synthesis and degradation generating diacylglycerol, a substrate for triglyceride synthesis, without affecting the expression of *SREBP1* gene.

Of note, while both studies found a remarkably consistent phenotype (i.e., increased hepatic triglyceride content), the mechanism leading to this phenotype was found to be different (Fig. 1). Xia *et al.* found that the increase in liver lipids was due to an increase in triglyceride synthesis mediated by SREBP-1c, while Tanaka *et al.* found that this increase was due to a novel non-canonical pathway supplying substrates from PI to triglycerides through a futile cycle. These differences may be partially explained by the different administration of the diet in the two studies. Indeed, in the study by Xia *et al.* (8), mice were fed a fasting-refeeding regime that is known to enhance the activation of SREBP pathway (10), whereas Tanaka *et al.* have used an ad libitum diet that does not affect this pathway. Perhaps the truth is somewhere in the middle, and both mechanisms contribute to the observed phenotype.

**Fig. 1:**
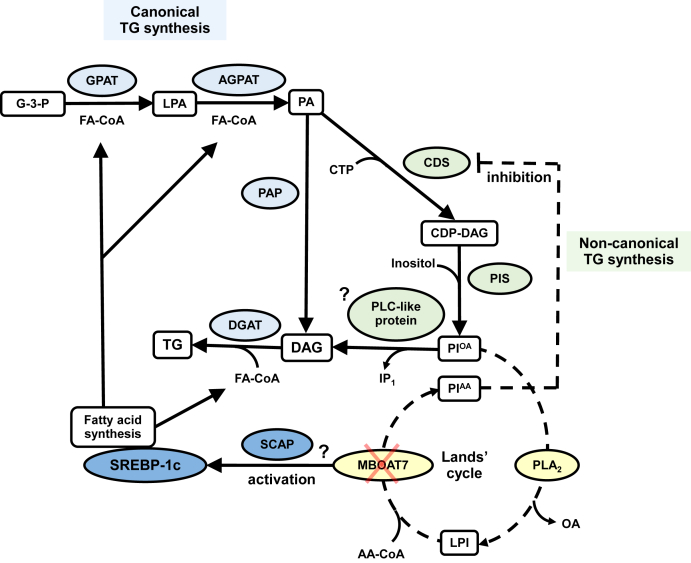
Molecular pathways by which *MBOAT7/LPIAT1* depletion induces hepatic steatosis. MBOAT7/LPIAT1 has an acyltransferase activity, incorporating arachidonic acid into lysophosphatidylinositol in the Lands' cycle. Xia *et al.* recently suggest that *MBOAT7* depletion promotes an increase in de novo lipogenesis driven by the activation of SREBP-1c. In an independent work, Tanaka *et al.* propose that *MBOAT7* depletion simultaneously causes an increased PI synthesis and PI degradation mediated by an unknown protein with PLC activity resulting in DAG, a substrate of triglyceride synthesis. These mechanisms contribute to the development of *MBOAT7*-induced hepatic steatosis, although they have yet to be entirely elucidated. AA, arachidonic acid; AGPAT, 1-acylglycerol-3-phosphate-O-acyltransferase; CDP-DAG, cytidine diphosphate diacylglycerol; CDS, cytidine diphosphate diacylglycerol synthase; DAG, diacylglycerol; DGAT, diacylglycerol O-acyltransferase; FA, fatty acids; G-3-P, glycerol-3-phosphate; GPAT, glycerol-3-phosphate acyltransferase; IP_1_, inositol monophosphate; LPA, lysophosphatidic acid; LPI, lysophosphatidylinositol; MBOAT7, membrane-bound O-acyltransferase domain–containing 7; OA, oleic acid; PA, phosphatidic acid; PAP, phosphatidate phosphatase; PI^AA^, phosphatidylinositol with arachidonic acid; PI^OA^, phosphatidylinositol with oleic acid; PIS, PI synthase; PLA_2_, phospholipase A2; PLC, phospholipase C; SCAP, SREBP cleavage–activating protein; SREBP-1c, sterol regulatory element–binding protein-1c; TG, triglyceride.

To conclude, Xia *et al.* robustly show that liver-specific *Mboat7* depletion causes an increase in liver fat content because of SREBP-1c–mediated increase in triglyceride synthesis. There are several other questions that remain to be solved: (a) what is the catalytic site of this protein, (b) how does *MBOAT7* depletion increase the susceptibility to liver inflammation, fibrosis, and hepatocellular carcinoma, and (c) last but not least, what is the mechanism by which the depletion of this gene causes liver and at the same time neurological disease. Further experimental studies are needed to answer these questions and to assess whether MBOAT7 may represent a novel pharmacological target(s) for NAFLD treatment in humans.

## Conflict of interest

The authors declare that they have no conflicts of interest with the contents of this article.
